# Commentary: Swapping or Dropping? Electrophysiological Measures of Difficulty during Multiple Object Tracking

**DOI:** 10.3389/fpsyg.2016.00372

**Published:** 2016-03-17

**Authors:** Błażej Skrzypulec

**Affiliations:** Department of Philosophy, Pontifical University of John Paul IIKraków, Poland

**Keywords:** visual sameness, multiple object tracking, contralateral delay activity (CDA), equivalence relations, object perception

Within cognitive psychology it is widely accepted that the human visual system represents the numerical sameness of objects. In particular, we are able to perceive an object as being the same despite movement and qualitative changes (Pylyshyn, 2007). Empirical research concerning visual sameness has focused on the conditions under which sameness is perceived (Odic et al., 2012) and the nature of mechanisms engaged in representing sameness (Makovski and Jiang, 2009).

However, the relation of visual sameness itself has not attracted as much attention and no detailed description of this relation is yet available. One of the most important questions is whether this relation can be understood as classical identity, and thus whether it is an equivalence relation, i.e., reflexive (object is the same as itself), symmetric (if A is the same as B, then B is the same as A), and transitive (if A is the same as B and B is the same as C, then A is the same as C).

While the topic of relations' formal properties is not completely alien to psychological research—see classical investigations by Tversky (1977) or more recent by Rips (2011)—there are no investigations that address the equivalence of visual sameness.

Despite this research gap, I intend to show that results of some psychological works can be interpreted as having a high relevance for the question of equivalence. I demonstrate this by analyzing a study by Drew et al. (2013) that is not explicitly concerned with the equivalence of visual sameness, but in fact has important implications for this question.

## Empirical test of equivalence

**Figure 1:**
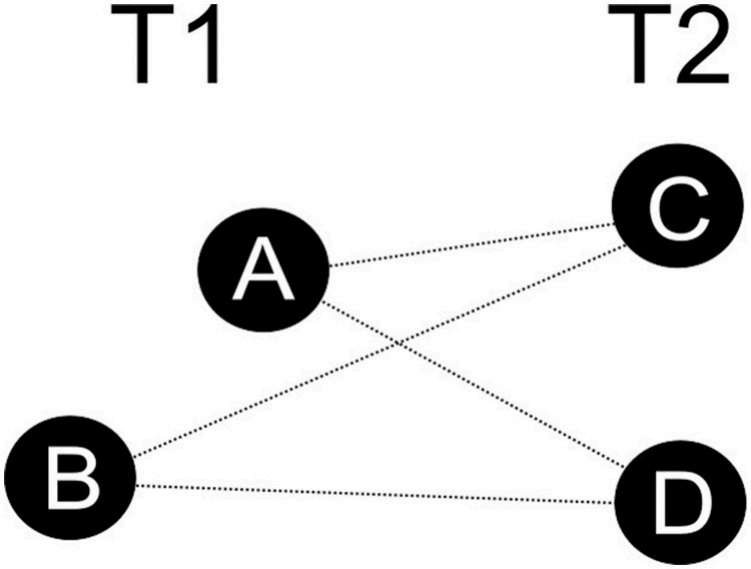
**A splitting-like situation**.

The equivalence of visual sameness can be empirically tested by investigating the behavior of the perceptual system in ambiguous splitting-like situations (see Figure 1). In such situations there is at least one object (A and B in Figure 1) at some moment T1 and at least two objects (C and D) at a subsequent moment T2. The pattern is ambiguous, since pairs A/C, A/D, B/C, B/D satisfy conditions that in ordinary circumstances are sufficient for representing objects as being the same, for example spatial cohesion and continuity in case of vision. On the other hand, the objects C and D are different in that they are spatially disconnected. If a visual system confronted with a splitting-like situation represents that the object A (or B) is the same as both objects at T_2_, then the sameness cannot be an equivalence relation and some non-classical model of sameness has to be adopted. If sameness were an equivalence relation, then objects C and D would also be the same due to symmetry and transitivity. However, in a splitting-like situation they are different objects.

It should be noted that it is not necessary for objects to spatially overlap in order to produce a splitting-like situation (as shown in Figure 1). What is sufficient, is the proximity between objects at subsequent moments. Tracking objects that are close to each other and predicting their future position relying on motion parameters is an effortful process which is prone to errors, especially when several objects are simultaneously tracked among distractors (Intriligator and Cavanagh, 2001; Pylyshyn, 2004; Keane and Pylyshyn, 2006). Because of that, in some cases of representing objects' close encounters, it may ambiguous how to establish sameness between objects at subsequent moments.

## Contralateral delay activity and visual sameness

In one of their experiments, Drew et al. (2013) applied the Multiple Object Tracking paradigm, in which participants are presented with a set of simple items having the same features. Initially, some of the presented objects were designated as targets, while the remaining ones played the role of distractors. Subsequently, all the objects started to move and the task of participants was to track the targets. After some time the objects stopped and participants were then asked to point out the targets. While the authors do not investigate the equivalence of visual sameness, the applied methodology allows for drawing a conclusion regarding this question.

One of the main sources of error in the re-identification of targets is the proximity between targets and distractors, since the number of errors is higher when objects meet more often (Bae and Flombaum, 2012). This source of error is relevant for the question of equivalence because when a target and a distractor are in proximity, a splitting-like situation is likely to occur: at T_1_ there are two objects, one target and one distractor, and at a subsequent moment T_2_ there are also two objects. In this case, the target from T_1_ may be represented as being the same as exactly one object at T_2_. Alternatively, sameness may not be represented between any objects at T_1_ and T_2_. Finally, it may be the case that the target at T_1_ is represented as being the same as both objects at T_2_. The occurrence of this last “double sameness” variant would constitute evidence against the hypothesis that visual sameness is an equivalence relation.

In the study by Drew et al. (2013), occurrences of re-identification errors were investigated by conducting electrophysiological measurements of Contralateral Delay Activity (CDA). According to earlier results, CDA is higher when the number of tracked targets is larger (Drew and Vogel, 2008).

This positive correlation allows us to evaluate how splitting-like situations are resolved. The “double-sameness” pattern should be associated with rising CDA: in a splitting-like situation there is one target at T_1_ but two targets at T_2_, as they are both represented as being the same as the target at T_1_. The growing number of targets leads to higher CDA.

In one of the experiments conducted by Drew et al. (2013: 215–216) the number of distractors was manipulated. The higher number of distractors should have led to more frequent errors caused by the proximity between objects. At least some of these errors may be interpreted as resulting from splitting-like situations. Yet such experimental design does not change other factors, such as objects' velocity, which may independently contribute to a higher number of errors. The investigations revealed that when the number of distractors was larger, there were more re-identification errors. However, the CDA remained constant, which suggests that the number of objects represented as targets did not change (Drew et al., 2013: 216–217). This result is consistent with the hypothesis that visual sameness is an equivalence relation.

## Conclusions

The formal properties of visual sameness can not only be empirically tested, some studies implicitly contain evidence relevant for evaluating the hypothesis that visual sameness is an equivalence relation. The study by Drew et al. (2013) is an important example, the results of which support the equivalence interpretation of sameness.

## Author contributions

The author confirms being the sole contributor of this work and approved it for publication.

## Funding

The work was supported by the National Science Center (Poland) grant 2014/12/T/HS1/00249.

### Conflict of interest statement

The author declares that the research was conducted in the absence of any commercial or financial relationships that could be construed as a potential conflict of interest.
